# Modulation of PDX1 gene expression and glycemic control by *Citrullus lanatus* in experimental type 2 diabetes

**DOI:** 10.1038/s41598-025-33982-9

**Published:** 2026-01-06

**Authors:** Anthony G. S. Moore, Stanley I. R. Okoduwa

**Affiliations:** 1 Department of Biochemistry, School of Basic Medical Sciences, Babcock University, Ilishan-Remo, Nigeria; 2 Department of Biological Sciences, Adventist University of West Africa, Advent Hill, Schiefflin Township Liberia; 3 Department of Research Innovation Management, Nigerian Institute of Leather and Science Technology, Zaria, Nigeria

**Keywords:** *Citrullus lanatus*, Type 2 diabetes, PDX1 gene expression, Pancreatic β-Cell function, Glycemic control, Oxidative stress, Biochemistry, Diseases, Endocrinology, Physiology

## Abstract

Diabetes mellitus is a major global health burden characterized by hyperglycemia, oxidative stress, and progressive pancreatic β-cell dysfunction. Despite the availability of conventional therapies, the need for safer and more effective alternatives persists. *Citrullus lanatus* (African watermelon) is a fruit rich in bioactive compounds with reported antihyperglycemic and antioxidant properties. This study investigated the modulatory effects of *C. lanatus* juice on pancreatic and duodenal homeobox 1 (PDX1) gene expression and glycemic control in fortified-diet-fed streptozotocin-induced diabetic Wistar rats. Thirty-six rats weighing 90–110 g, were randomized into six groups: normal control (NMC), diabetic control (DBC), metformin-treated diabetic rats (DBM, 500 mg/kg), and diabetic rats treated with *C. lanatus* juice at 200 mg/kg (DBCL1), 500 mg/kg (DBCL2), and 1000 mg/kg (DBCL3). Animals were pre-fed either normal or fortified diets prior to diabetes induction with streptozotocin (35 mg/kg). Following 14 days of treatment, blood and pancreatic tissues were analyzed for glycemic, antioxidant, and molecular parameters. *C. lanatus* juice significantly (*p* < 0.05) reduced fasting blood glucose in a dose-dependent manner, with the greatest effect observed in DBCL3. Antioxidant enzymes (catalase, superoxide dismutase, and glutathione peroxidase) were significantly increased, while malondialdehyde levels were reduced in all treated groups. Notably, PDX1 gene expression was markedly upregulated (*p* < 0.05) in *C. lanatus*-treated rats, accompanied by improved insulin, C-peptide, and HDL levels. These findings suggest that *C. lanatus* juice exerts antidiabetic effects that are associated with increased PDX1 mRNA expression, improved glycemic control, and reduced oxidative stress, supporting a potential role for transcriptional modulation of β-cell function, highlighting its potential as a functional dietary intervention for type 2 diabetes management.

## Introduction

Diabetes mellitus remains one of the foremost causes of global morbidity and mortality. It contributes significantly to complications such as cardiovascular diseases, renal failure, blindness, and lower limb amputations^1,2^. According to the International Diabetes Federation (IDF) Diabetes Atlas, 2025, approximately 643 million adults (aged 20–79 years) are currently living with diabetes worldwide, and this number is projected to rise to 783 million by 2045. The highest growth is expected in low- and middle-income countries, particularly in sub-Saharan Africa, where prevalence is estimated to increase by over 129% by 2045. These alarming statistics underscore the urgent need for innovative therapeutic and preventive strategies to mitigate the global diabetes burden^3^.

Type 2 diabetes mellitus (T2DM) accounts for more than 90% of all diabetes cases and is characterized by insulin resistance, impaired insulin secretion, and progressive β-cell dysfunction. It does not segregate between race, age, gender or status of the individual^4,5^. Chronic hyperglycemia, oxidative stress, and lipotoxicity contribute to the decline in β-cell mass and insulin production^6^. Sustained high glucose and fatty acids promote insulin resistance, decreased pancreas mass, and dysfunctional insulin^7,8^. Central to maintaining β-cell identity and insulin gene transcription is the pancreatic and duodenal homeobox 1 (PDX1) gene—a key transcription factor whose expression is suppressed under diabetic oxidative conditions. PDX1 downregulation has been linked to impaired insulin biosynthesis and β-cell apoptosis, making it a potential therapeutic target in T2DM management^9^.

The PDX1 gene is a protein encoding gene recognised for its regulatory role in the development and maintenance of pancreatic β-cell identity^10,11^. As a potential target for T2DM, PDX1 gene expression is significantly down-regulated by oxidative stress^12^. Oxidative stress downregulation in the management of T2DM has now gained more research focus and attention. Current T2DM drugs, including metformin, with adverse side effects, remained prominent in the face of the yet-unresolved increase in T2DM cases. This led researchers to seek out natural alternatives.

Despite the availability of synthetic antidiabetic agents like metformin, adverse effects and the rising incidence of diabetes have sparked interest in plant-derived therapeutics^13^. Natural products rich in antioxidants have shown promise in attenuating oxidative stress and restoring pancreatic function. *C. lanatus* (African watermelon), a fruit abundant in bioactive compounds like lycopene and citrulline, has been previously reported to possess antihyperglycemic and antioxidant properties. However, its potential to modulate PDX1 gene expression remains largely unexplored.

Studies have identified bioactive compounds from plants for their possible ameliorating in vivo and in vitro prospects in antidiabetic activities^13^. Lycopene and citrulline, found abundantly in *Citrullus lanatus*, have been shown to possess antihyperglycemic and antilipidemic properties^14–17^. *C. lanatus*, a tropical to moderate climate fruit, has over the centuries served many therapeutic purposes, including anticancer, anti-inflammation, anti-vascular diseases, and anti-diabetes^17–19^. In light of these discoveries, the anti-diabetic activities of *C*. *lanatus* on PDX1 gene expression have not been well explored. The PDX1 gene, a key transcriptional regulator of pancreatic β-cell identity, has been identified as a prospective molecular target in T2DM, although functional outcomes depend on regulation at multiple levels beyond mRNA expression.

Although numerous studies have established the antioxidant and antihyperglycemic activities of *C. lanatus*, there is limited understanding of its molecular mechanism of action, particularly concerning genes involved in β-cell function and insulin biosynthesis^9,12,20,21^. Oxidative stress is a central contributor to the development and complications of diabetes mellitus. Excessive production of reactive oxygen species (ROS) and inadequate antioxidant defense disturb cellular redox balance, resulting in lipid peroxidation, protein oxidation, and DNA damage. In pancreatic β-cells, which possess inherently low antioxidant capacity, oxidative stress leads to mitochondrial dysfunction, apoptosis, and impaired insulin secretion. In peripheral tissues, it contributes to insulin resistance by disrupting insulin receptor signaling and glucose uptake mechanisms. Consequently, regulating oxidative stress has become a vital therapeutic target in preventing β-cell failure and metabolic complications associated with diabetes (Robertson, 2020; Matough et al., 2022).

Beyond its metabolic consequences, oxidative stress also influences gene regulatory networks crucial for β-cell identity and function. Elevated ROS suppress the transcriptional activity of PDX1, a master regulator of insulin gene expression and β-cell maintenance. Mechanistically, oxidative stress activates stress-responsive pathways such as JNK and FoxO1, leading to PDX1 nuclear exclusion, post-translational modification, and degradation. Sustained downregulation of PDX1 under these conditions results in β-cell dysfunction and apoptosis, contributing to progressive hyperglycemia. Understanding the association between oxidative stress and altered PDX1 transcription provides a strong rationale for investigating antioxidant interventions such as *C. lanatus* juice in diabetes management^12,20,21^. The PDX1 gene is a critical transcription factor responsible for maintaining β-cell identity and regulating insulin gene expression. Downregulation of PDX1 under diabetic oxidative conditions contributes to β-cell dysfunction and apoptosis. Despite this, no previous study has examined whether *C. lanatus* can modulate PDX1 expression. Addressing this gap, the present study explores the potential of *C. lanatus* juice to modulate PDX1 gene expression and related β-cell functional markers, thereby providing insight into possible molecular associations, offering new mechanistic insight into its antidiabetic action.

This study, therefore, investigates the effect of *C. lanatus* juice on PDX1 gene expression and glycemic control in fortified diet-fed streptozotocin-induced diabetic Wistar rats. By evaluating antioxidant status, lipid profiles, and β-cell functional markers, the study aims to assess whether *C. lanatus* can serve as a functional dietary intervention for T2DM through molecular and metabolic modulation.

## Materials and methods

### Plant materials

The plant *C. lanatus* (Thunb.) Matsum. and Nakai (watermelon) was purchased from Ilishan-Remo Market, Ogun State, Nigeria, which were all obtained directly from a single local farm during the same harvest period (to minimize variability in watermelon composition due to cultivar and seasonal factors), and taken to the University of Ibadan, Oyo State, Nigeria, for botanical identification and authentication with the Forest Herbarium, Ibadan (FHI). The voucher specimen No. 113,954 was deposited. The fruits were processed under uniform laboratory conditions to ensure chemical consistency and reproducibility. The reddish-pink, watery pulp of *C. lanatus* was sliced, and the seeds removed. Four litres of juice were extracted using an electric blender and oven-dried at 50 ℃ to obtain a semisolid paste (215.32 g). The drying step effectively reduced moisture and enzyme activity, enhancing product stability. The paste was then stored at 10–20 ℃ in airtight containers, a condition under which previous studies have shown minimal degradation of bioactive compounds^14,16^. Although refrigeration at 4 ℃ is typically optimal, the paste remained stable at 10–20 ℃ due to its low water content and short-term storage period.

Phytochemical characterization of the *C. lanatus* juice extract was not performed in this study. Consequently, no assumptions are made regarding the presence, concentration, or relative abundance of specific phytochemical constituents in the extract used. References to previously reported constituents of *C. lanatus* are provided solely for contextual background and do not constitute compositional confirmation of the extract evaluated in this work that contribute to its antioxidative and antihyperglycemic properties^14,16,19^. These established findings provided the biochemical rationale for evaluating its potential modulation of PDX1 gene expression. Future studies will incorporate GC–MS or HPLC-based profiling to identify and quantify the specific bioactive constituents responsible for the observed effects.

### Experimental animals

The acute toxicity of *C. lanatus* juice was not re-assessed in this study, as prior investigations have demonstrated its high safety margin. Studies by Ajiboye et al.^22^ and Jibril et al.^23^ reported no mortality or adverse behavioral changes in Wistar rats administered up to 5000 mg/kg of *C. lanatus* extracts or juice. Given its wide traditional dietary use and previously confirmed non-toxic profile, the present study focused on evaluating its antidiabetic and molecular effects rather than acute toxicity testing. To closely replicate the pathophysiology of human type 2 diabetes, this study employed a combination of a fortified (high-fat/fructose) diet to induce insulin resistance and a low-dose STZ injection to trigger partial β-cell dysfunction. This dual model effectively captures the metabolic and pancreatic impairments characteristic of T2DM, providing a more physiologically relevant framework for assessing the therapeutic effects of *C. lanatus*^24^.

Animals comprising 36 male Wistar rats weighing 90–110 g, were obtained from the Babcock University Animal Facility. These animals were acclimatised for two weeks in a well-ventilated room at 20–25 °C, with 12 h of daylight and 12 h of darkness (OECD and WHO laboratory standards). During this period, animals were given normal rat feed and tap water. After the acclimatisation period, animals were assigned to two feeding groups: normal diet-fed (NDF) and fortified diet-fed (FDF). This protocol was adopted from the work of Okoduwa et al.^24^. Normal diet-fed (crude protein 16.0% min, fat 3.0% min, fibre 8.0% max, calcium 1.0% min, available phosphorus 0.4% min, and energy 2600 kcal/kg min), and fortified diet-fed (NDF, margarine, plus 20% fructose drink). The FDF was in the ratio of 5 g of NDF to 1 g of Simas margarine (fat 99.9%, emulsifier E471, E322, antioxidant E304, E306, beta-carotene C175130, Vitamin A 30,000 IU/kg, Vitamin D 30,000 IU/kg). After five weeks of a dietary regimen and a 14-day *C. lanatus* administration, animals were sacrificed by cervical dislocation without prior anaesthesia and/or analgesia, minimising potential chemical interference and suffering for the animals^25^. For biochemical evaluation, terminal blood from a cardiac puncture and pancreatic tissue were obtained.

### Ethical considerations

Ethical approval for the study was obtained from the Babcock University Health Research Ethics Committee (BUHREC 048/24). All methods were carried out in accordance with the relevant guidelines and regulations of the institution and national standards for the care and use of laboratory animals. Furthermore, all methods are reported in accordance with the ARRIVE guidelines (https://arriveguidelines.org) for animal research. All experimental procedures were conducted in compliance with the Nigerian National Code of Health Research Ethics (NNCHRE, 2019) and the institutional guidelines of the University Animal Research Ethics Committee (UAREC/2024/012). Animals were humanely sacrificed by cervical dislocation performed by trained personnel without prior anesthesia, as permitted under local and OECD guidelines for small rodents when executed rapidly and skillfully to minimize pain and distress. The method was approved as part of the ethical clearance for this study.

### Chemicals and reagents

Streptozotocin was purchased from Nanjing Forever Pharmacy Co., Ltd. (Nanjing City, China). Fructose (MOLYCHEM, India), Simas Margarine (PT Salim Ivomas Pratama Tbk, Indonesia), Normal Diet Feed (Animal Care Services Konsult Ltd., Nigeria); Lipid profile and oxidative stress biomarkers kits (Randox Laboratories, UK); ultra-sensitive rat insulin ELISA kit (Elabscience Biotechnology Inc., USA); rat C-peptide ELISA kit (Elabscience Biotechnology Inc., USA); RNA extraction kits (Direct-zol RNA MiniPrep, Zymo Research, USA); Primers and reagents (Inqaba Biotec West Africa Ltd., Nigeria); and all other reagents were of analytical grade and purchased from recognised and credible companies and laboratories in Nigeria.

### Equipment

On Call Plus glucometer and strips (ACON Biotech Hongzhou Co., Ltd., China). Real-time polymerase chain reaction (RT-PCR) machine for gene expression analysis (LineGene 9600 Plus Fluorescent Quantitative Detection System, BIOER). An electronic weighing scale, hot-air oven, incubator, ELISA microplate reader, and microscope were among the items of equipment used.

### Induction of diabetes

After five weeks of a diet regimen, animals were fasted overnight for 10–12 h, weighed, and fasting blood glucose measured. All experimental animals, except the normal control, were administered 35 mg/kg per body weight of streptozotocin in a citrate buffer with a pH of 4.5 intraperitoneally and were provided a 5% glucose solution in the first 24 h.

The combination of fortified diet feeding and STZ administration (35 mg/kg) was used to establish a reliable model of type 2 diabetes, as the diet induces insulin resistance while STZ causes partial β-cell damage. This approach produces a stable diabetic phenotype resembling human T2DM, as previously validated by Okoduwa et al.^24^.

### Diabetes confirmation and validation

Confirmation and validation of diabetes were done after 72 h and one week of post-STZ induction, respectively^24^. Fasting blood glucose was measured using a glucose metre and strips. Rats considered diabetic (> 200 mg/dl fasting blood glucose or > 300 mg/dl non-fasting blood glucose) were selected for the study^24^. All 36 rats survived the STZ induction and treatment periods, with no mortality recorded. Mild, transient hyperglycemic symptoms (polyuria and lethargy) were observed within 48 h post-induction, confirming successful diabetes establishment. All animals resumed normal activity before treatment initiation.

### Experimental design

Thirty-six (36) experimental animals weighing 90–110 g, were randomly assigned to six groups (*n* = 6): normal control (no diabetes, no treatment), negative control (diabetes, no treatment), positive control (diabetes, 500 mg/kg metformin-treated), experimental test 1 (diabetes, 200 mg/kg watermelon juice-treated), experimental test 2 (diabetes, 500 mg/kg watermelon juice-treated), and experimental test 3 (diabetes, 1000 mg/kg watermelon juice-treated). The doses of 200, 500, and 1000 mg/kg body weight of *C. lanatus* juice were selected based on previously reported efficacious ranges in diabetic Wistar rats^22,23^. These doses also align with traditional use levels when scaled from human consumption. Furthermore, the doses have been shown to produce significant improvements in glycemic control and antioxidant parameters without inducing toxicity. A preliminary laboratory screening further confirmed their safety and dose-dependent biological activity, justifying their use for evaluating both metabolic and molecular responses in this study.

Animals were treated for two weeks. The treatment period of 14 days was selected in accordance with previous studies demonstrating that *C. lanatus* and other phytotherapeutic interventions elicit significant glycemic and molecular responses within 10–21 days of administration in rodent models of type 2 diabetes^22,23^. This duration was deemed adequate to evaluate the acute modulatory effects on PDX1 gene expression and oxidative stress markers. However, longer-term studies are warranted to assess the sustained antidiabetic efficacy and potential cumulative effects of *C. lanatus* juice.

### Observation of experimental animals

Daily fluid and feed intake were measured using a measuring cylinder and an electronic weighing scale, respectively. The animals were weighed weekly.

### Biochemical analysis

*Fasting blood glucose*.

The fasting blood glucose (FBG) level was measured using a glucose metre and strips. Before and after 72 h of STZ induction, after a week of STZ induction, and subsequently, weekly FBG measured during the two weeks of treatment. Blood was obtained from animals’ tails.

*Lipid profile determination*.

Total cholesterol, high-density lipoprotein (HDL), and triglycerides were analysed using Randox kits in accordance with the manufacturer’s manual. Low-density lipoprotein (LDL) was determined from values obtained from total cholesterol and HDL. These assays were carried out using animals’ serum.

*Serum insulin determination*.

The serum insulin and C-peptide levels were determined using ultra-sensitive rat insulin and rat C-peptide ELISA kits, according to the manufacturer’s procedures.

*Homeostatic model assessment (HOMA) score*.

The scores for insulin resistance and beta-cell function (HOMA-IR and HOMA-β) were calculated using the formula described by Matthews et al.^26^.

*Measurement of oxidative stress biomarkers*.

Malondialdehyde (MDA), catalase (CAT), superoxide dismutase (SOD), and glutathione peroxidase (GPx) were measured using procedures outlined in their respective assay kits. Animals’ pancreatic tissues were used in analysing the oxidative stress biomarkers.

*Pancreatic duodenal homeobox-1 gene analysis*.

Primers by Babaiedarzi et al.^27^, which matched the primers designed, were used. Total RNA was extracted from the pancreas using RNA extraction kits following the manufacturer’s procedures. The extracted RNA was quantified using a nanodrop spectrophotometer at A260/A280 nm. A one-step real-time polymerase chain reaction was carried out to evaluate the level of PDX-1 gene expression. PDX1 expression was assessed at the mRNA level only; protein expression and functional activity were not evaluated in this study.

### Histopathological analysis

Pancreatic tissues were dehydrated by immersing them in ascending concentrations of alcohol solutions (70–100%) and in paraffin. Slices of 4–5 μm of pancreas slides were prepared and stained with hematoxylin and eosin (H&E). Slides were analysed under a light microscope at 100X and photographed with a Zeiss Axio photomicroscope.

### Statistical analysis

All statistical analyses were performed using SPSS version 21, Excel 2019, and GraphPad Prism 10. Data were expressed as mean ± standard deviation (SD). Group differences were evaluated using one-way analysis of variance (ANOVA). Following one-way ANOVA, post hoc multiple comparison testing was performed using Duncan’s Multiple Range Test (DMRT) for biochemical and oxidative stress parameters and Tukey’s test for gene expression analyses. Superscript letters in tables denote statistically homogeneous subsets, where identical letters indicate no significant difference and different letters indicate significant difference at *P* < 0.05.

## Results and discussion

### Pretreatment feed and fluid consumption

**Fig. 1:**
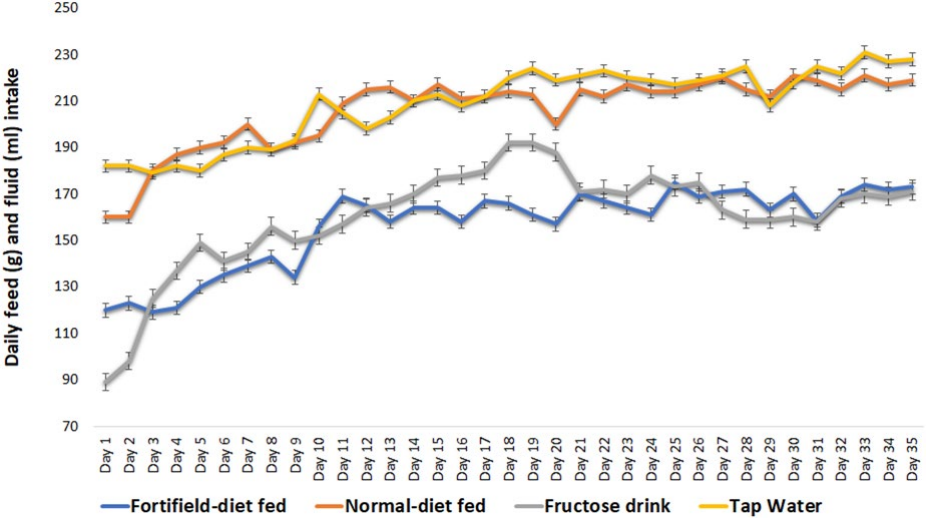
Daily feed and fluid intake of dietary regimen prior to induction with Streptozotocin.

During the pretreatment period with normal diet feed (NDF) and fortified diet feed (FDF), animals showed higher consumption of NDF and tap water compared to FDF and fructose drink (Fig. 1). This difference may be attributed to the higher palatability of the NDF compared with the fat-enriched fortified diet^24^. Consumption gradually increased and stabilized by day 21. Previous findings suggest that sugar and salt are more effective in promoting feed intake than lipid enrichment^28^.

Daily feed and fluid intake are presented as “mean ± SD” (*n* = 6).

### Change in animals’ weight, feed and fluid intake

**Fig. 2:**
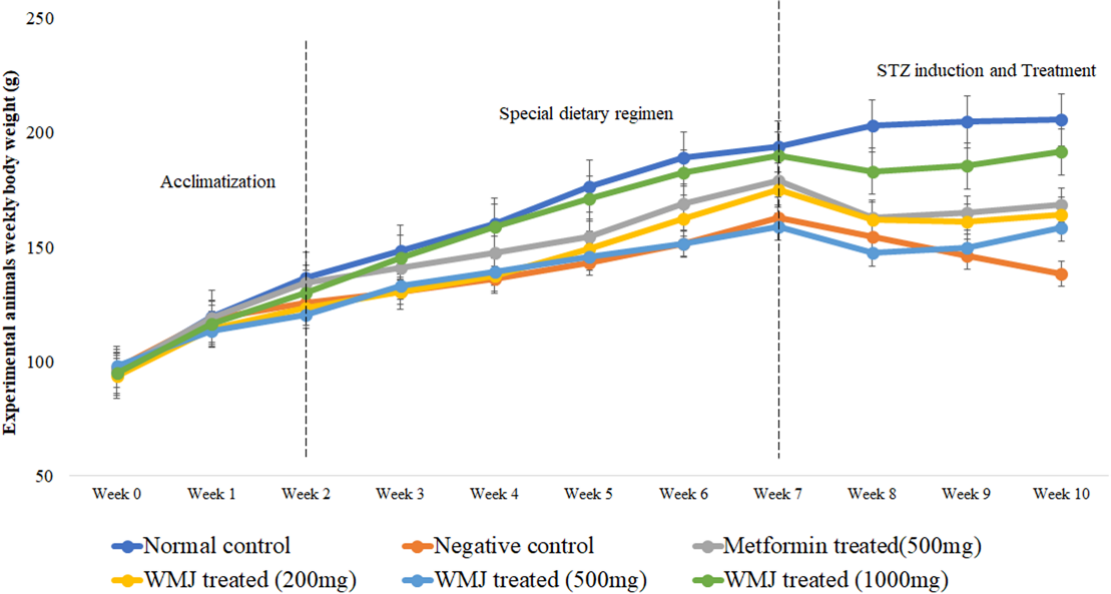
Weekly change in body weight of experimental animals.

**Fig. 3:**
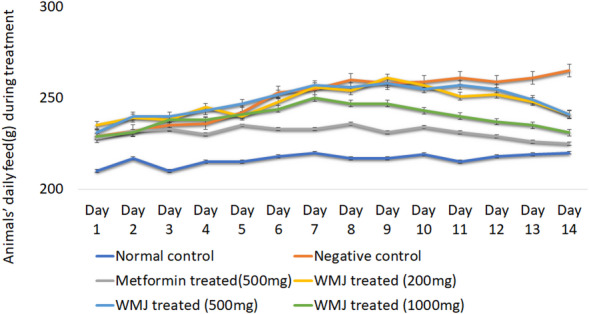
Daily feed intake during treatment for each experimental group.

**Fig. 4:**
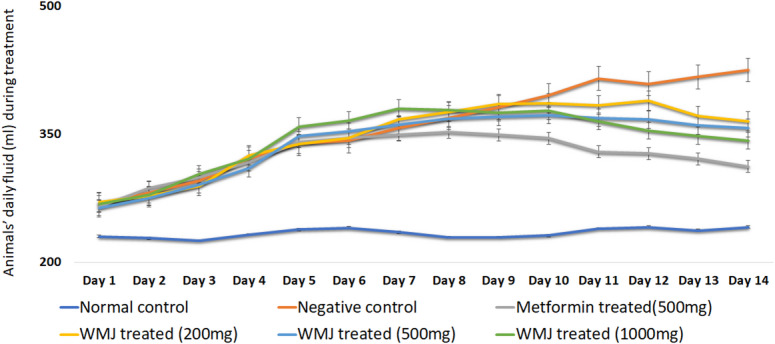
Daily fluid intake during treatment for each experimental group.

Body weight remained comparable between NDF and FDF groups prior to induction (Fig. 2). One week after STZ induction, a significant weight reduction and increase in feed and fluid intake were observed (Figs. 2, 3 and 4), confirming hyperglycemic symptoms characteristic of diabetes^29^. Treatment with C. lanatus juice for 14 days gradually normalized feed and fluid intake and increased body weight, suggesting improvement in glycemic control and energy metabolism, consistent with earlier reports^23^.

All animals were fed normal diet feed during acclimatisation (weeks 0–2). At weeks 3 through 10, animals were divided into two feeding groups (normal-diet-fed and fortified-diet-fed). The data is represented by the mean ± SD of each experimental group, with *n* = 6. Watermelon juice (WMJ).

Daily feed intake by six groups of experimental animals after streptozotocin induction at week 8 and through week 10 is presented as “mean ± SD” (*n* = 6). Watermelon juice (WMJ).

Daily fluid intake by six groups of experimental animals after streptozotocin induction at week 8 and through week 10 is presented as “mean ± SD” (*n* = 6). Watermelon juice (WMJ).

### Effects of *C. lanatus* on fasting blood glucose

Following STZ induction, fasting blood glucose (FBG) levels increased significantly in all diabetic groups. After 14 days of treatment, fasting blood glucose levels decreased significantly (*p* < 0.05) in all *C. lanatus*-treated groups in a clear dose-dependent pattern (Tables 1 and 2). This reduction aligns with previous reports that *C. lanatus* juice and leaf extracts improve glycemic control by enhancing insulin secretion and reducing oxidative stress in diabetic rodents^22,23^. The dose-dependent pattern observed here further indicates that the *C. lanatus* juice extract may enhance β-cell responsiveness. The observed normalization of glucose levels in the 1000 mg/kg group suggests an improvement in β-cell functional status, which may be associated with increased PDX1 gene expression, although direct mechanistic links require further validation.

**Table 1:** Fasting blood glucose of experimental animals at selected stages following streptozotocin (STZ) induction (35 mg/kg body weight) in all diabetic groups, including the negative control.

Groups	FBG Prior to Induction (mg/dl)	FBG 72 h Post-Induction	FBG 1 Week Post-Induction (mg/dl)	FBG 1 Week Treatment (mg/dl)	FBG 2 Weeks Treatment (mg/dl)
Normal control	82.17 ± 2.99^a, b^	82.00 ± 2.37^a^	80.00 ± 1.41^a^	81.67 ± 2.34^a^	80.67 ± 1.63 ^a^
Negative control (STZ-induced, untreated)	82.33 ± 1.21^a, b^	330.33 ± 19.05^c^	255.67 ± 12.80^b^	276.33 ± 25.26^e^	281.50 ± 17.33^e^
Metformin treated (500mg)	85.17 ± 1.94^b^	280.00 ± 16.30^b^	276.83 ± 21.18^c^	156.83 ± 13.47^b^	102.17 ± 7.08^b^
WMJ treated (200mg)	81.83 ± 3.55^a, b^	302.17 ± 44.12^b^	250.00 ± 4.94^b^	227.50 ± 3.62^d^	198.17 ± 3.76^d^
WMJ treated (500mg)	84.00 ± 0.89^a, b^	365.83 ± 4.02^d^	262.67 ± 6.65^b^	231.50 ± 3.39^**d**^	178.83 ± 6.97^c^
WMJ treated (1000mg)	81.17 ± 1.17^a^	298.83 ± 22.05^b^	252.00 ± 5.90^b^	177.17 ± 0.98^c^	109.67 ± 2.07^b^

Values are expressed as mean ± SD (*n* = 6). Within each column, values sharing the same superscript letter are not significantly different, whereas values with different superscript letters differ significantly at *P* < 0.05, as determined by one-way ANOVA followed by post hoc multiple comparison testing. Watermelon juice (WMJ).

Fasting blood glucose (FBG) was recorded at the major stages of the experiment:

1. After five weeks of a dietary regimen at week 7;
2. After 72 h of streptozotocin (STZ) induction;
3. One-week post-STZ induction at week 8;
4. One-week treatment at week 9; and.
5. Two-week treatment at week 10.

**Table 2:** Percent change in FBG at treatment with *C. lanatus* juice.

Groups	FBG 1 Week Post Induction (mg/dl)	1 Week	2 Weeks
		% Change	% Change
Normal control	80.00 ± 1.41^a^	2.04 ± 0.03^e^	0.83 ± 0.92^e^
Negative control (STZ-induced, untreated)	255.67 ± 12.80^b^	7.48 ± 0.34^f^	9.18 ± 0.12^f^
Metformin treated (500 mg)	276.83 ± 21.18^c^	− 76.51 ± 0.35^a^	− 170.96 ± 0.13^a^
WMJ treated (200 mg)	250.00 ± 4.94^b^	− 9.89 ± 0.08^d^	− 26.15 ± 0.028^d^
WMJ treated (500 mg)	262.67 ± 6.65^b^	− 13.46 ± 0.33^c^	− 46.88 ± 0.11^c^
WMJ treated (1000 mg)	252.00 ± 5.90^b^	− 42.24 ± 0.17^b^	− 129.79 ± 0.09^b^

Percent change in fasting blood glucose (FBG) recorded at each week of treatment, for a total of two weeks. The negative (-ve) sign before a number means a decrease in FBG. Watermelon juice (WMJ). Values are expressed as mean ± SD (*n* = 6). Within each column, values sharing the same superscript letter are not significantly different, whereas values with different superscript letters differ significantly at *P* < 0.05, as determined by one-way ANOVA followed by post hoc multiple comparison testing.

### Effect of *C. lanatus* juice on serum insulin and C-peptide of experimental animals

**Fig. 5:**
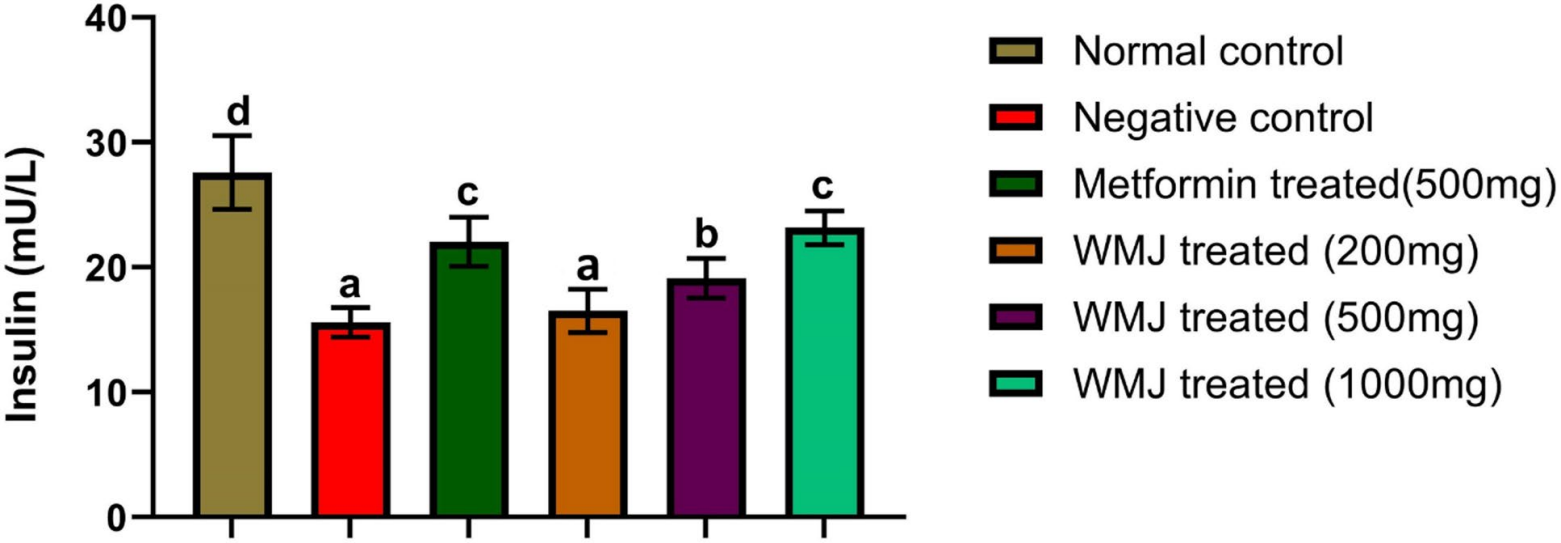
Serum insulin level of experimental animals.

**Fig. 6:**
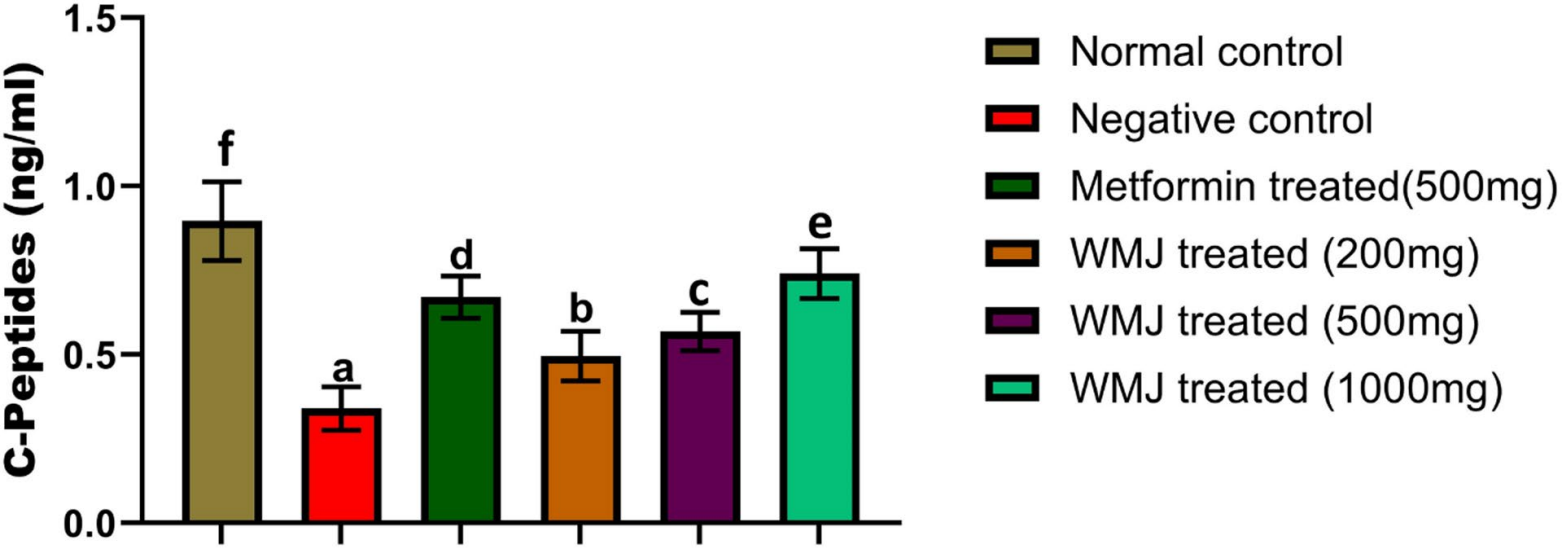
Serum C-peptides level in experimental animals.

*C. lanatus* treatment improved insulin and C-peptide levels in a dose-dependent manner (Figs. 5 and 6). The 500 mg/kg and 1000 mg/kg groups exhibited significantly higher insulin levels compared to the diabetic control. These findings are consistent with reports that *C. lanatus* enhances insulin secretion through antioxidant protection of β-cells and modulation of key metabolic genes^22,30^. The concurrent rise in C-peptide further confirms improved endogenous insulin synthesis rather than external modulation.

The data represents the mean ± SD of all experimental groups. The different letters attached to each bar indicate a significant difference with *p* < 0.05. (*n* = 6). Watermelon juice (WMJ).

The data represents the mean ± SD of all experimental groups (*n* = 6). The different letters attached to each bar indicate a significant difference with *p* < 0.05. Watermelon juice (WMJ).

### Effects of *C. lanatus* on insulin resistance and β-cell function

Homeostasis model assessment of insulin resistance (HOMA-IR) and homeostasis model assessment for beta-cell function (HOMA-β) results are recorded in Table 3. *C. lanatus* juice-treated groups showed significant (*p* < 0.05) beneficial effects on beta-cell function in a dose-dependent manner. Also, insulin resistance was significantly (*p* < 0.05) decreased in all *C. lanatus-*treated groups compared with the negative control group. Animals’ fasting blood glucose after two weeks of treatment with *C. lanatus* juice and animals’ terminal insulin levels were used in deriving HOMA scores^26,31^. Findings are indicative of type-2 diabetes, but none of these (HOMA-IR or HOMA-β) alone is sufficient to predict prediabetes and diabetes^32,33^. Also, *C. lanatus* was effective in reducing insulin resistance and improving beta-cell function in STZ-induced diabetes rats.

**Table 3:** Quantification of insulin resistance and β-cell function in experimental animals.

Groups	FBG (mg/dl)	HOMA-IR	HOMA-β
Normal control	80.67 ± 1.63^a^	5.50 ± 0.42^a^	119.67 ± 0.23^f^
Negative control (STZ-induced, untreated)	281.50 ± 17.33^e^	10.84 ± 0.11^c^	16.45 ± 0.32^a^
Metformin treated (500 mg)	102.17 ± 7.08^b^	5.56 ± 1.03^a^	74.18 ± 0.13^e^
WMJ treated (200 mg)	198.17 ± 3.76^d^	8.08 ± 0.57^b^	26.51 ± 0.48^b^
WMJ treated (500 mg)	178.83 ± 6.97^c^	8.44 ± 0.31^b^	34.99 ± 0.01^c^
WMJ treated (1000 mg)	109.67 ± 2.07^b^	6.27 ± 0.19^a^	72.55 ± 0.32^d^

Values are expressed as mean ± SD (*n* = 6). Within each column, values sharing the same superscript letter are not significantly different, whereas values with different superscript letters differ significantly at *P* < 0.05, as determined by one-way ANOVA followed by post hoc multiple comparison testing. Fasting blood glucose (FBG), homeostasis model assessment of insulin resistance (HOMA-IR), homeostasis model assessment for beta-cell function (HOMA-β), and watermelon juice (WMJ).

### Effects of *C. Lanatus* juice on serum lipid profile of experimental animals

*C. lanatus* administration improved serum lipid profiles in diabetic rats (Table 4). HDL increased while LDL, triglycerides, and total cholesterol decreased in a dose-dependent manner. The 1000 mg/kg group showed lipid levels approaching those of metformin-treated animals. These findings corroborate previous studies reporting lipid-lowering and cardioprotective properties of *C. lanatus*^34^. The mechanism likely involves enhanced antioxidant activity, leading to reduced lipid peroxidation and improved lipid metabolism^35^.

**Table 4:** Effects of *C. lanatus* juice on serum lipid profile of experimental animals.

Groups	High density lipoprotein (mmol/l)	Low density lipoprotein (mmol/l)	Triglycerides (mmol/l)	Cholesterol (mmol/l)
Normal control	2.53 ± 0.25^e^	0.94 ± 0.06^a^	3.96 ± 1.64^a^	4.63 ± 0.63^a^
Negative control (STZ-induced, untreated)	0.41 ± 0.13^a^	18.78 ± 1.22^f^	35.12 ± 1.45^f^	23.41 ± 1.51^f^
Metformin treated (500 mg)	1.42 ± 0.04^c^	4.18 ± 0.85^c^	12.98 ± 0.06^d^	11.14 ± 1.25^c^
WMJ treated (200 mg)	0.98 ± 0.09^b^	9.06 ± 0.09^e^	18.91 ± 0.06^e^	17.85 ± 1.07^e^
WMJ treated (500 mg)	1.53 ± 0.30^c^	5.48 ± 0.23^d^	11.87 ± 0.37^c^	12.98 ± 1.28^d^
WMJ treated (1000 mg)	2.17 ± 0.25^d^	1.35 ± 0.36^b^	8.82 ± 0.81^b^	8.34 ± 0.95^b^

Values are expressed as mean ± SD (*n* = 6). Within each column, values sharing the same superscript letter are not significantly different, whereas values with different superscript letters differ significantly at *P* < 0.05, as determined by one-way ANOVA followed by post hoc multiple comparison testing. Watermelon juice (WMJ).

### Effect of *C. Lanatus* on oxidative stress in pancreatic tissues of experimental animals

Oxidative stress markers revealed substantial differences among groups (Table 5). STZ-induced diabetic rats showed elevated MDA and decreased antioxidant enzyme levels (CAT, SOD, GPx), confirming oxidative damage. *C. lanatus* juice significantly reversed these effects in a dose-dependent manner, restoring antioxidant defense comparable to metformin. Reduced levels of catalase, superoxide dismutase, and glutathione peroxidase, along with higher levels of malondialdehyde, were indicative of significant oxidative stress in the negative control group^13,36^. On the other hand, *C. lanatus* juice administration improved oxidative stress biomarkers, indicative of its antioxidative qualities, as previously noted^18,22^. It can be said that *C. lanatus* has antioxidative actions necessary for ameliorating oxidative stress in STZ-induced diabetic rats.

**Table 5:** Level of oxidative stress on pancreatic tissues in experimental animals.

Groups	Catalase (U/mg)	Superoxide dismutase (U/ml)	Glutathione Peroxidase (U/l)	Malondialdehyde (µM)
Normal control	39.89 ± 2.38^f^	3.66 ± 0.04^f^	3.16 ± 0.07^f^	0.40 ± 0.05^a^
Negative control (STZ-induced, untreated)	6.28 ± 0.69^a^	1.54 ± 0.28^a^	0.59 ± 0.07^a^	4.39 ± 0.11^e^
Metformin treated (500 mg)	26.83 ± 0.54^d^	2.64 ± 0.02^c^	2.27 ± 0.12^c^	0.79 ± 0.13^c^
WMJ treated (200 mg)	18.84 ± 0.19^b^	2.45 ± 0.19^b^	1.94 ± 0.07^b^	1.48 ± 0.07^d^
WMJ treated (500 mg)	25.25 ± 0.91^c^	2.80 ± 0.24^d^	2.54 ± 0.08^d^	0.74 ± 0.07^c^
WMJ treated (1000 mg)	34.99 ± 2.45^e^	3.20 ± 0.04^e^	3.07 ± 0.03^e^	0.49 ± 0.04^b^

Values are expressed as mean ± SD (*n* = 6). Within each column, values sharing the same superscript letter are not significantly different, whereas values with different superscript letters differ significantly at *P* < 0.05, as determined by one-way ANOVA followed by post hoc multiple comparison testing. Watermelon juice (WMJ).

### Effects of *C. Lanatus* juice on PDX1 gene expression in experimental animals

**Fig. 7:**
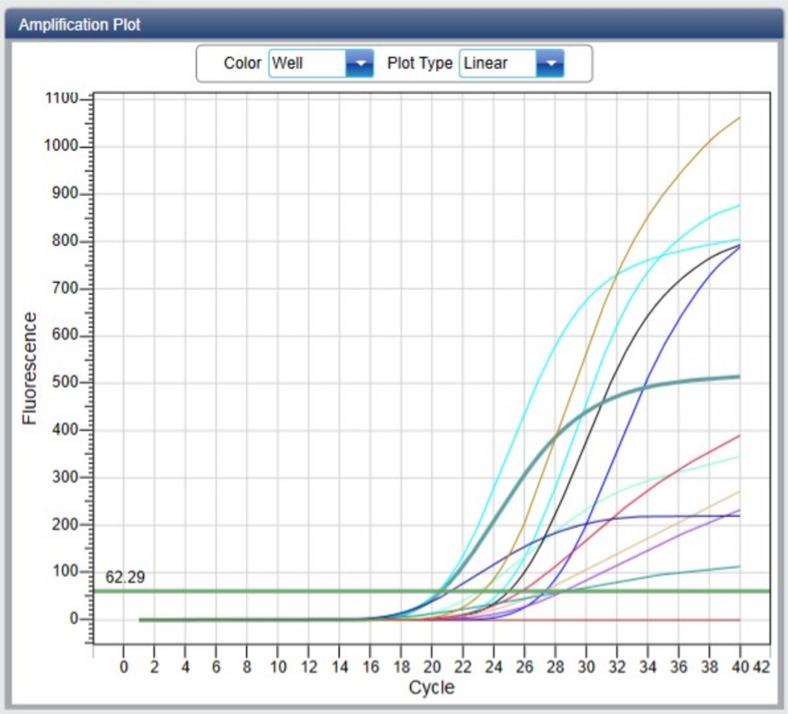
Diagram showing PDX1 gene analysis with real-time polymerase chain reaction.

**Fig. 8:**
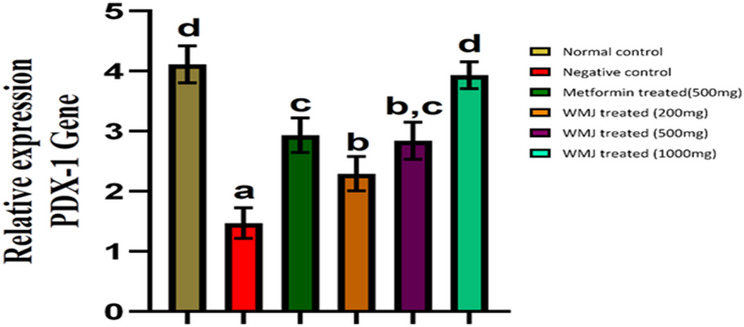
Effects of *C. Lanatus* juice on PDX1 gene expression in the pancreas of experimental animals.

*C. lanatus* juice significantly upregulated PDX1 gene expression in a dose-dependent manner (Figs. 7 and 8). The highest increase occurred in the 1000 mg/kg group, approaching normal control levels. STZ-induced rats showed marked PDX1 suppression, consistent with oxidative stress–mediated β-cell dysfunction. Decreased expression of the PDX1 gene was significantly (*p* < 0.05) seen in the negative control group. The increase in PDX1 mRNA expression following *C. lanatus* treatment suggests a transcriptional response consistent with improved β-cell integrity, although this does not directly confirm protein-level restoration or β-cell regeneration. This aligns with studies linking antioxidant therapy to preservation of β-cell transcription factors^20,37,38^. A quantitative study also confirmed the relationship between elevated oxidative stress and type 2 diabetes as well as hypertension^39^.

The observed upregulation of PDX1 mRNA expression following C. lanatus treatment may reflect transcriptional responses associated with the administration of the crude juice extract. While antioxidant-related mechanisms are plausible, definitive attribution to specific phytochemical constituents cannot be made in the absence of extract-level quantitative characterization, which are known to exert potent antioxidant and cytoprotective effects. Oxidative stress has been shown to downregulate PDX1 expression through suppression of pancreatic transcriptional activity and activation of apoptotic cascades^20^. Lycopene, a carotenoid abundant in *C. lanatus*, can activate the Nrf2/Keap1 signaling pathway, enhancing the expression of endogenous antioxidant enzymes (SOD, CAT, GPx), thereby protecting β-cells from oxidative DNA and protein damage. Similarly, citrulline supports nitric oxide homeostasis and mitochondrial function, reducing oxidative burden and sustaining PDX1 transcriptional activity. These observations suggest that the increased PDX1 mRNA expression observed in this study may reflect antioxidant-mediated modulation of redox-sensitive transcriptional pathways, although confirmation at the protein and signaling levels is required, contributing to β-cell regeneration and improved insulin synthesis^9,13,16,20^.

The observed upregulation of PDX1 mRNA expression aligns with emerging evidence that antioxidant interventions can counteract oxidative suppression of β-cell transcription factors, primarily at the transcriptional level^9,16^. These recent findings collectively support our hypothesis that *C. lanatus* bioactives act via redox-sensitive transcriptional regulation pathways.

Pancreatic duodenal homeobox 1 (PDX1) gene expression is shown here by a real-time polymerase chain reaction (RT-PCR) graph. The Y axis is plotted with the frequencies of the fluorescence dye. The X axis is plotted with the number of RT-PCR cycles per gene expressed. The lower the cycle number above the threshold (62.29), the higher the number of DNA in solution.

The data represent the mean ± SD of each experimental group (*n* = 6). The letters attached to the bar indicate a significant difference (*p* < 0.05). Watermelon juice (WMJ) and pancreatic duodenal homeobox 1 (PDX1).

### Histopathological effects of *C. Lanatus* juice on the pancreas of experimental animals

**Fig. 9:**
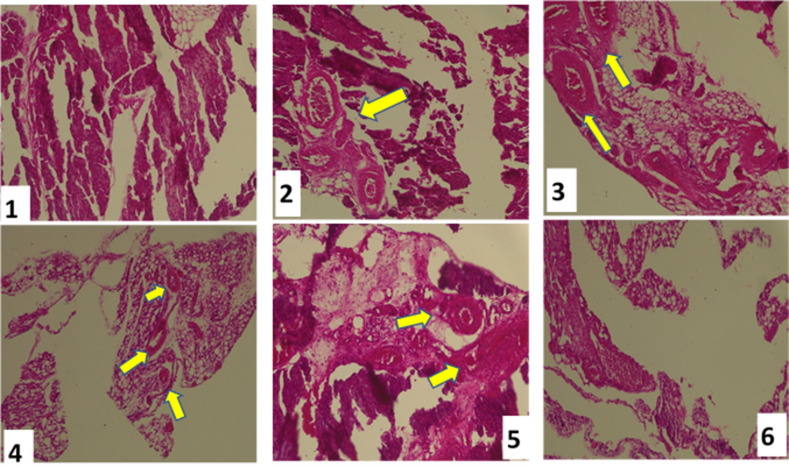
Representative histopathological sections of pancreatic tissues (H&E stain). Yellow arrows indicate islet lesions and necrotic β-cells. Images illustrate overall pancreatic and islet architecture across experimental groups. Due to image resolution and magnification constraints, the figure is intended for qualitative comparison only and does not support quantitative evaluation of β-cell mass or cellular morphology. (1) Normal control; (2) Diabetic control; (3) Metformin-treated (500 mg/kg); (4–6) *C. Lanatus* juice–treated groups (200, 500, and 1000 mg/kg), showing progressive regeneration and improved cellular architecture. *Images are presented at low magnification to illustrate overall pancreatic architecture.

Histopathological examination of pancreatic sections revealed qualitative differences in overall islet architecture among experimental groups (Fig. 9). Diabetic control rats exhibited distorted pancreatic morphology with apparent islet disruption compared to normal controls, whereas *C. lanatus*–treated groups showed partial preservation of general islet structure. However, due to the absence of high-magnification images and quantitative morphometric analysis, these observations are limited to descriptive, low-resolution structural features and do not permit detailed assessment of β-cell mass, shrinkage, or cellular regeneration. These structural improvements corroborate biochemical and molecular findings, indicating that *C. lanatus* mitigates oxidative injury and supports β-cell structural preservation, with concurrent increases in PDX1 mRNA expression^30^.

Taken together, these findings demonstrate that *C. lanatus* juice effectively ameliorates hyperglycemia, dyslipidemia, and oxidative stress in STZ-induced diabetic rats through both biochemical and molecular mechanisms. The observed restoration of PDX1 expression and preservation of pancreatic histoarchitecture suggest a potential β-cell–protective role of *C. lanatus*. These integrated results form the basis for the concluding remarks presented in this study.

The histopathological findings presented in this study are qualitative and supportive in nature. In the absence of high-magnification imaging and quantitative histological scoring, pancreatic histology was not used to infer β-cell mass, cellular regeneration, or precise architectural remodeling. Instead, histology was included to provide contextual structural observations that complement the biochemical and transcriptional findings.

A limitation of the present study is the absence of quantitative phytochemical characterization of the *C. lanatus* juice extract. Plant-derived products are known to exhibit batch-to-batch variability in phytochemical composition due to factors such as cultivar, maturity, processing, and storage conditions. Therefore, while prior studies have identified various bioactive compounds in *C. lanatus*, the present findings should be interpreted strictly at the level of the administered crude extract. Future studies incorporating HPLC-, LC–MS-, or GC–MS–based profiling are required to identify, quantify, and standardize the constituents responsible for the observed biological and transcriptional effects. A limitation of this study is the absence of higher-magnification histopathological images (40× or 100×), which restricted detailed cellular-level assessment of pancreatic islet morphology. Consequently, histological interpretations were based on overall structural preservation rather than fine β-cell ultrastructure. Future studies incorporating higher-resolution imaging and quantitative morphometric analysis would strengthen histopathological evaluation.

Another limitation of the present study is that only PDX1 mRNA expression was analyzed as a molecular endpoint. While this provides preliminary evidence of transcriptional modulation, further validation through upstream (FoxO1) and downstream (GLUT2, INS) gene analysis would better define the regulatory network underlying β-cell protection. Moreover, protein-level confirmation of PDX1 expression through Western blot or immunohistochemical analysis would strengthen the mechanistic interpretation of the observed effects. Future studies are therefore planned to integrate these approaches to provide a comprehensive understanding of how *C. lanatus* bioactives modulate β-cell function and glycemic regulation.

A further limitation of this study is that molecular interpretation was based solely on PDX1 mRNA expression. While changes in mRNA levels provide valuable insight into transcriptional regulation, they do not necessarily reflect corresponding changes in protein abundance, localization, or functional activity. Consequently, the present findings should be interpreted as supportive of transcriptional modulation rather than definitive mechanistic evidence. Future studies incorporating protein-level validation (e.g., Western blotting or immunohistochemistry) and analysis of upstream and downstream signaling pathways are required to confirm the functional role of PDX1 in mediating the observed β-cell effects.

## Conclusion

This study demonstrates that *C. lanatus* juice significantly ameliorates hyperglycemia, dyslipidemia, and oxidative stress while upregulating PDX1 gene expression in STZ-induced type 2 diabetic rats. These effects suggest that *C. lanatus* may support pancreatic β-cell function and insulin biosynthesis, with effects associated with antioxidant activity and increased PDX1 gene expression, although definitive molecular mechanisms require further investigation. However, as these findings were obtained from an animal model, they should be interpreted with caution when considering human relevance. Differences in metabolism, dosage scaling, and long-term responses between species limit direct extrapolation. Therefore, further studies—including isolation of active compounds, mechanistic exploration in human cell models, and controlled clinical trials—are required to validate the therapeutic potential of *C. lanatus* juice in human diabetes management. Nonetheless, this study provides novel mechanistic insight into the role of *C. lanatus* in modulating PDX1 expression and oxidative stress, offering a strong foundation for future translational research.

## Data Availability

The datasets used and/or analysed during the current study are available from the first author on reasonable request.
